# Homologous Double-Antibody Sandwich ELISA Targeting a Linear Epitope of FPV VP2 and Demonstration of Microtubule-Dependent Intracellular Trafficking

**DOI:** 10.3390/microorganisms14102412

**Published:** 2026-10-21

**Authors:** Shi-Jun Zhang, Yang Wang, Geng-Yi Hu, Jing-Yang Li, Wei-Fei Lu, Chao Zhang, Jia-Jia Pan, Qing-Yong Guo, Lei Zeng

**Affiliations:** 1 College of Veterinary Medicine, Henan Agricultural University, Zhengzhou 450046, Chinachaofirst1@163.com (C.Z.); first2015@163.com (J.-J.P.); 2 Key Laboratory of Animal Biochemistry and Nutrition, Ministry of Agriculture and Rural Affairs, Zhengzhou 450046, China; 3 Key Laboratory of Veterinary Biotechnology of Henan Province, Henan Agricultural University, Zhengzhou 450046, China; 4 College of Animal Science and Technology, Xinjiang Agricultural Vocational and Technical University, Changji 831100, China; 5 College of Veterinary Medicine, Xinjiang Agricultural University, Urumqi 830052, China

**Keywords:** feline panleukopenia virus, VP2, monoclonal antibody, DAS-ELISA, microtubule transport

## Abstract

Feline panleukopenia (FP) is a highly fatal disease in cats caused by feline panleukopenia virus (FPV). The viral capsid protein VP2 determines immunogenicity and host range. We isolated an FPV strain, ZZ202501, and generated four monoclonal antibodies (mAbs) against VP2 by immunizing mice with purified virus and screening the resulting hybridomas on recombinant VP2 expressed in *Escherichia coli*, a strategy that biases selection toward linear denaturation-resistant epitopes. One mAb, 4D10, bound a linear denaturation-resistant epitope and gave the highest signal-to-noise ratio among the 16 capture and detection combinations tested, which allowed it to serve as both capture and detection antibody in a double-antibody sandwich ELISA (DAS-ELISA). The assay detected 10^1.67^ TCID_50_/0.1 mL of virus and did not cross-react with feline calicivirus or feline herpesvirus. Across 136 clinical samples, it showed complete agreement with a conventional PCR assay. Using mAb 4D10, we also followed the intracellular distribution of the total VP2 pool during infection. VP2 underwent a time-dependent cytoplasmic-to-nuclear redistribution that was blocked by nocodazole, an inhibitor of microtubule polymerization, and microtubule disruption reduced progeny virus titer by more than 100-fold without detectable cytotoxicity. These data indicate that microtubule-dependent trafficking contributes to efficient FPV replication. The study provides an analytically characterized DAS-ELISA for FPV antigen detection, together with evidence linking cytoskeletal transport to viral propagation, and illustrates why the choice between linear and conformational epitopes matters in assay design.

## 1. Introduction

Feline panleukopenia virus (FPV), classified by the International Committee on Taxonomy of Viruses within the species Protoparvovirus carnivoran1 of the family Parvoviridae [1], causes a systemic disease in cats characterized by fever, vomiting, diarrhea, dehydration and a marked fall in circulating leukocytes [2,3]. Morbidity and mortality are highest in kittens and in unvaccinated animals [4], and the World Organization for Animal Health lists feline panleukopenia among the notable infectious diseases in cats [5,6]. Since its recognition in the early twentieth century, the virus has become globally distributed [7]. Inactivated and live-attenuated vaccines are in widespread use, yet clinical cases continue to be reported in vaccinated animals [8], and antigenic variants arising from substitutions in the capsid gene complicate control. FPV remains prevalent in China, North America, Europe and Southeast Asia [9,10]. The major capsid protein VP2 mediates receptor binding and carries the dominant antigenic determinants of the virus [11,12]. Infection is directed principally at hematopoietic precursors in the bone marrow and at the intestinal crypt epithelium, and the host antibody response is largely directed against VP2 [13]. Because substitutions at key residues of VP2 can reduce the reactivity of diagnostic antibodies and the cross-protection afforded by vaccines, assays directed at conserved regions of VP2 are of practical value.

Methods available for the detection of FPV [14], include virus isolation, TaqMan probe-based multiplex real-time PCR [15], SYBR Green real-time PCR [16], immunohistochemistry (IHC) [17], loop-mediated isothermal amplification [18,19], enzyme-linked immunosorbent assay (ELISA) [20,21] and colloidal gold immunochromatography [22,23]. Most target VP2. As the virus accumulates substitutions, assays that depend on a particular VP2 conformation or on a narrowly defined epitope may fail to detect variant strains, and the type of epitope recognized by the detecting antibody therefore deserves more attention than it usually receives. Antibodies raised against native conformational epitopes are well suited to capturing intact virions [24,25], but their reactivity depends on the capsid remaining assembled, which cannot be assumed for fecal samples subjected to transport and storage. Antibodies directed at linear epitopes that survive denaturation retain reactivity with both assembled and partially degraded antigen.

To obtain such an antibody we immunized mice with purified virus but screened the resulting hybridomas on recombinant VP2 expressed in *Escherichia coli*, which lacks eukaryotic folding and post-translational modification and therefore selects for antibodies whose epitopes survive denaturation. One antibody obtained in this way, mAb 4D10, recognized a denaturation-resistant epitope and functioned as both capture and detection reagent in a homologous-pairing DAS-ELISA. We consider that this behavior may reflect repeated display of the epitope on the assembled capsid, although the epitope was not mapped in this study, and the interpretation remains to be tested. Because 4D10 detects VP2 irrespective of assembly state, we also used it to follow the total intracellular VP2 pool during infection and found that its redistribution to the nucleus requires an intact microtubule network and is associated with efficient production of progeny virus. The antibody described here may thus be useful both for antigen-detection assays and for studies of VP2 trafficking.

## 2. Materials and Methods

### 2.1. Viruses, Cells, Sera, and Animals

The FPV ZZ202501 strain was isolated in our laboratory from FPV-positive clinical specimens and was used throughout this study. FPV-positive sera were taken from the sample collection held in our laboratory; positivity had been established by PCR amplification of the VP2 gene with Sanger confirmation of the amplicon, and the sera originated from separate clinical cases rather than from experimental infection. Crandell–Rees feline kidney (CRFK) cells were purchased from Wuhan Nuopusai Life Technology Co., Ltd. (Wuhan, China). Murine myeloma SP2/0 cells were obtained from the American Type Culture Collection (ATCC, Manassas, VA, USA). CRFK cells were maintained in Minimum Essential Medium (MEM; Gibco, 02026, Thermo Fisher Scientific, Waltham, MA, USA) with 10% fetal bovine serum (FBS; Gibco, 10099, USA) at 37 °C in 5% CO_2_. SP2/0 cells were maintained in serum-free hybridoma medium (BasalMedia, H630KJ, Shanghai, China) at 37 °C in 5% CO_2_. *Escherichia coli TOP10* was held in our laboratory, and E. coli BL21 (DE3) was purchased from Beijing Tsingke Biotechnology Co., Ltd. (Beijing, China). Six-week-old specific-pathogen-free female BALB/c mice were purchased from the Laboratory Animal Center of Zhengzhou University (Zhengzhou, China); three were used for immunization and hybridoma production and three for ascites production. Fecal samples and rectal swabs were suspended in PBS, vortexed for 1 min and centrifuged at 10,000× *g* for 10 min at 4 °C, and the clarified supernatant was used directly for ELISA and for nucleic acid extraction. All animal procedures were approved by the Institutional Animal Care and Use Committee of Henan Agricultural University (Approval No. HNND2025040812) and were carried out in accordance with the Guide for the Care and Use of Laboratory Animals and the relevant regulations of Henan Agricultural University.

### 2.2. FPV Propagation, Purification and Concentration

CRFK cells were seeded into culture flasks and, at 80% confluence, inoculated with the FPV ZZ202501 isolate at a multiplicity of infection (MOI) of 0.1. After adsorption for 1 h, the inoculum was replaced with MEM containing 2% FBS, and the cells were incubated at 37 °C in 5% CO_2_ until the cytopathic effect (CPE) involved approximately 90% of the monolayer. Flasks were then subjected to two or three freeze–thaw cycles, and the lysate was centrifuged at 8000× *g* for 10 min. The supernatant was collected and passed through a 0.22 μm filter. For purification, the clarified supernatant was concentrated by ultracentrifugation at 100,000× *g* for 2 h, the pellet was resuspended in PBS, and the suspension was purified on an iodixanol density gradient. For sensitivity testing, a portion of the purified virus was concentrated a further ten-fold on a 100 kDa ultrafiltration device (Millipore, Burlington, MA, USA) to give a high-titer stock. Infectious titers were determined on CRFK cells in 96-well plates by ten-fold serial dilution with eight replicate wells per dilution, with the CPE being recorded after 5 to 7 days, and were calculated by the Reed–Muench method and expressed as TCID_50_/0.1 mL. For plaque purification, CRFK monolayers were infected with serial dilutions of virus, overlaid with MEM containing 1% low-melting-point agarose and incubated for 5 to 7 days; individual plaques were picked with sterile pipette tips, carried through three successive rounds of plaque purification and amplified in CRFK cells.

### 2.3. Preparation of Recombinant FPV VP2 Protein and Its Use as a Screening Antigen

Specific primers (Table S1) were designed based on the reference genome sequence of FPV (GenBank accession number: M38246.1). Using the purified FPV obtained in Section 2.2 as the template, the *VP2* gene was amplified by PCR. The amplified VP2 fragment was cloned into the pET-21b-NusA expression vector using homologous recombination after digestion of the vector with BamHI and XhoI. The recombinant plasmid was verified by sequencing and designated pET-21b-NusA-FPV-VP2. The confirmed recombinant plasmid was subsequently transformed into *Escherichia coli* *BL21 (DE3)* competent cells. For recombinant VP2 expression, the transformed bacterial cultures were grown until the optical density at 600 nm (OD600) reached 0.5–0.8. Protein expression was induced by adding 0.5 mM isopropyl β-D-1-thiogalactopyranoside (IPTG), followed by incubation overnight at 18 °C with shaking at 180 rpm. The bacterial cells were collected and disrupted, and the recombinant VP2 protein was purified from the bacterial lysate using Ni-NTA affinity chromatography with an imidazole gradient of 25–500 mM. The purified protein was analyzed by SDS-PAGE. Because prokaryotic expression precludes eukaryotic post-translational modifications and folding chaperones, the resulting recombinant VP2 is largely denatured/linearized. Using it as the coating antigen in indirect ELISA biases hybridoma screening toward mAbs recognizing linear denaturation-resistant epitopes, which are more stable in degraded clinical samples and remain detectable even when native capsid conformation is disrupted.

### 2.4. Animal Immunization Strategy and Selection of Positive Hybridomas

Three BALB/c mice were immunized using the purified live FPV suspension as the immunogen. For the primary immunization, 100 μL of the virus suspension was emulsified with an equal volume (100 μL) of Freund’s complete adjuvant and administered via subcutaneous multi-point injection into the back. Three weeks later, a second immunization was performed via intraperitoneal injection using 100 μL of the virus suspension emulsified with 100 μL of Freund’s incomplete adjuvant. A third immunization was administered two weeks later, using the same dose and route as the second immunization. One week after the final immunization, blood samples were collected from the tail vein, and the serum was separated to evaluate the antibody titer by indirect ELISA using the recombinant VP2 protein as coating antigen.

The mouse exhibiting the highest antibody titer was selected for spleen collection and subsequent cell fusion experiments. Briefly, a single-cell suspension was prepared from the mouse spleen. Polyethylene glycol (PEG) 1450 was added to facilitate the fusion of SP2/0 myeloma cells with spleen cells at a ratio of 1:10. The fused cells were then seeded into 96-well plates containing HAT selection medium and incubated at 37 °C in a 5% CO_2_ atmosphere. Approximately seven days post-fusion, positive hybridoma cells were screened using an indirect ELISA coated with the recombinant VP2 protein. Positive clones were subsequently subcloned four times by limiting dilution. Finally, the monoclonal cell lines specifically secreting antibodies against the FPV VP2 protein were selected, expanded, and cryopreserved.

### 2.5. Indirect ELISA Procedure

Conditions for hybridoma screening were established by checkerboard titration with purified recombinant FPV VP2 as the coating antigen and sera from FPV-immunized and unimmunized mice as positive and negative controls. Recombinant VP2 was serially diluted from 800 to 50 ng/mL in coating buffer (0.1 mol/L carbonate buffer, pH 9.6) and the sera from 1:400 to 1:51,200. Conditions were selected on the criteria that the positive serum gave an OD450 above 1.0, the negative serum an OD450 below 0.2, and the positive-to-negative (P/N) ratio was maximal. On this basis, the coating concentration was set at 50 ng/mL and the serum dilution at 1:800 (Table S4). For screening of hybridoma supernatants, 96-well plates were coated overnight at 4 °C with recombinant FPV VP2 at 50 ng/mL, washed five times with PBST and blocked with 5% skimmed milk (200 μL per well) for 2 h at room temperature. After five further washes, 50 μL per well of post-fusion hybridoma supernatant was added and incubated for 1 h at room temperature, with sera from unimmunized and FPV-immunized mice as negative and positive controls. After five washes, horseradish peroxidase (HRP)-conjugated goat anti-mouse IgG (50 μL per well) was added and incubated for 1 h at room temperature. Plates were washed five times, and 100 μL per well of 3,3′,5,5′-tetramethylbenzidine (TMB) substrate was added. After development in the dark at room temperature, the reaction was stopped with 50 μL per well of stop solution, and the optical density was read at 450 nm on a microplate reader. The concentration of 3 μg/mL given in the originally submitted manuscript was an error and does not correspond to the condition used.

### 2.6. Western Blot

The protein samples were separated by SDS-PAGE and subsequently transferred onto a polyvinylidene fluoride (PVDF) membrane at a constant current of 250 mA for 30 min. The membrane was blocked with 5% skim milk for 2 h at room temperature. Following blocking, the membrane was incubated overnight at 4 °C with the culture supernatants from the screened positive monoclonal hybridomas. Sera from unimmunized mice or negative hybridoma culture supernatants were utilized as negative controls, while sera from FPV-immunized mice served as the positive control. After five washes with TBST, the membrane was incubated with horseradish peroxidase-conjugated goat anti-mouse IgG for 1 h at room temperature. The membrane was washed five times again with TBST, and the targeted protein bands were visualized using an enhanced chemiluminescence detection system. For the time-course expression analysis, HRP-conjugated anti-GAPDH monoclonal antibody (Proteintech, Rosemont, IL, USA, 60004-1-Ig, 1:5000 dilution) was used to detect GAPDH as the internal loading control.

### 2.7. Immunofluorescence Assay (IFA)

CRFK cells were infected with FPV. At 24 h post-infection (hpi), the culture supernatant was discarded, and the cells were fixed with 4% paraformaldehyde for 30 min. Subsequently, the cells were washed three times with phosphate-buffered saline (PBS) and permeabilized with 0.1% Triton X-100 for 15 min, followed by another three washes with PBS. Each well was then blocked with 5% skim milk (200 μL) for 1 h and washed three times with PBS. The screened monoclonal antibodies were applied as the primary antibodies and incubated for 1 h at room temperature. Following three washes with PBS, the cells were incubated with DyLight 488-conjugated goat anti-mouse IgG as the secondary antibody and then washed three times with PBS. Finally, the cell nuclei were counterstained with 4′,6-diamidino-2-phenylindole (DAPI), and the fluorescence signals were observed using a fluorescence microscope.

### 2.8. Preparation of Mouse Ascites and Purification of Monoclonal Antibodies

We cultivated the hybridoma cells to logarithmic growth stage and injected them into the peritoneal cavity of three female mice at a dose of 1 × 10^6^ (suspended in 500 μL) per mouse. These mice had been pretreated with Freund’s incomplete adjuvant one week prior to the injection. Ascitic fluid was collected aseptically 7 to 14 days post-injection. The collected fluid was centrifuged at 8000 rpm for 10 min to separate the clear ascites from the upper lipid layer and the lower cell pellet. Subsequently, the ascitic fluid was filtered through a 0.45 μm membrane and purified using the saturated ammonium sulphate precipitation method, followed by Protein G affinity chromatography.

### 2.9. Chromosome Number Analysis of Monoclonal Hybridoma Cells

Chromosome number analysis was performed on SP2/0 cells and selected monoclonal hybridoma cells. Cells were seeded in 6 cm dishes at 2 × 10^6^ cells/dish and treated with 0.05 μg/mL colchicine for 6 h when approximately 60% confluent. Cells were then collected and subjected to hypotonic treatment with prewarmed 4% KCl at 37 °C for 25 min followed by 25 min at room temperature. After centrifugation, cells were sequentially fixed with methanol:glacial acetic acid (3:1 and 1:1, *v*/*v*) at room temperature. The fixed cells were resuspended in 1 mL fixative, dropped onto precooled glass slides, and air-dried. The slides were stained with Giemsa solution for 2 min, rinsed with distilled water and 20% ethanol, and air-dried. Metaphase chromosome spreads were examined and photographed under a light microscope using a 40× objective lens, and chromosome numbers were determined by counting individual chromosomes in well-spread metaphase cells.

### 2.10. Tracking the Total VP2 Protein Pool During Infection

Cell coverslips were placed into 24-well plates, and CRFK cells were seeded at a density of 1 × 10^5^ cells/well. Upon reaching 50% confluence, the cells were infected with the FPV ZZ202501 strain at an MOI of 1. The cell coverslips were harvested at 0, 6, 12, 24, 36, and 48 hpi. The cells were subjected to IFA as described in Section 2.7, utilizing the mAb 4D10 (clone FPV-9D-4D-10B, diluted 1:400) as the primary antibody. For Western blot analysis, cells from parallel wells were lysed at the same time points. The lysates were separated by SDS-PAGE and subjected to immunoblotting as described in Section 2.6, with GAPDH serving as the loading control. Because mAb 4D10 recognizes a linear epitope, it detects all forms of VP2—free monomers, assembly intermediates, and mature capsids—thus revealing the total intracellular distribution of the VP2 protein pool.

### 2.11. Functional Analysis of Microtubule Inhibition on VP2 Trafficking and Viral Replication

To examine the contribution of the microtubule network to the subcellular localization of VP2, CRFK cells were seeded at 1 × 10^5^ cells per well into 24-well plates fitted with coverslips. At 50% confluence, the cells were pretreated for 4 h with MEM containing 20 μM nocodazole to depolymerize microtubules, with the control receiving an equivalent volume of DMSO. Cells were then infected with FPV ZZ202501 at an MOI of 1. After 1 h of adsorption, the medium was replaced with maintenance medium containing 20 μM nocodazole, and samples were harvested at 0, 6, 12 and 24 hpi. For immunofluorescence, the murine anti-VP2 mAb 4D10 (1:400) and a rabbit anti-α-tubulin monoclonal antibody (Cell Signaling Technology, Danvers, MA, USA, 2125, 1:500) were used as primary antibodies, with Alexa Fluor 488-conjugated goat anti-mouse IgG and Alexa Fluor 594-conjugated goat anti-rabbit IgG (1:1000) as secondary antibodies, and nuclei were counterstained with DAPI for 10 min. The distribution of VP2 and of microtubules was recorded on a laser scanning confocal microscope. Infectious titers in the culture supernatants of nocodazole- and DMSO-treated cultures were determined in triplicate at each time point by TCID_50_ assay on CRFK cells. To establish whether any reduction in titer could be attributed to loss of viable cells, CRFK cells were treated in parallel with 20 μM nocodazole or DMSO for 24 h, matching the duration of the infection experiment, and the viability was measured by Cell Counting Kit-8 assay in six replicate wells in three independent experiments.

### 2.12. Establishment of the Double-Antibody Sandwich ELISA

Conditions for the double-antibody sandwich ELISA (DAS-ELISA) were optimized by checkerboard titration. All 16 combinations of the four mAbs as capture and HRP-conjugated detection antibody were evaluated. Coating buffers were compared (carbonate buffer, 50 mM, pH 9.6; PBS, 10 mM, pH 7.4; Tris-HCl, 50 mM, pH 8.5; with deionized water as control), as were BSA and skimmed milk as blocking agents at 0.5%, 1%, 2.5%, 5% and 10%. All incubations were carried out at 37 °C with constant shaking. Working conditions were chosen on the criteria that the positive control gave an OD450 of at least 1.0, the negative control an OD450 of no more than 0.1, and the P/N ratio was maximal. These criteria served to select reagent concentrations and were not used to classify samples; the threshold applied to samples is defined in Section 2.14. Since VP2 is present in many copies in the parvovirus capsid, we considered that an antibody directed at a linear epitope might act as both capture and detection reagent and tested this by comparing all homologous and heterologous combinations.

### 2.13. Sensitivity, Specificity, and Reproducibility

A high-titer FPV ZZ202501 stock was prepared by ten-fold concentration of purified virus, giving a titer of 10^5.67^ TCID_50_/0.1 mL, and used as starting material. The analytical sensitivity was determined on ten-fold serial dilutions of this stock, from 10^5.67^ to 10^0.67^ TCID_50_/0.1 mL, tested by the established DAS-ELISA. The analytical specificity was assessed by testing the FPV-positive sample alongside samples positive for feline herpesvirus (FHV) and feline calicivirus (FCV). Canine parvovirus was not tested, as isolates were not available; the assay has therefore not been examined for discrimination between feline and canine parvoviruses. Intra-assay and inter-assay reproducibility were determined in triplicate and expressed as the coefficient of variation (CV).

### 2.14. Clinical Sample Testing and PCR Detection

A total of 136 clinical samples, comprising rectal swabs and fecal samples, were collected from cats with signs consistent with FPV infection and were tested both by the established DAS-ELISA and by a conventional PCR assay read on agarose gels. Real-time PCR was not used; so, cycle threshold values are not available for these samples. The PCR used primers directed at a conserved region of the *VP2* gene, FPV-*VP2*-F (5′-TGGTGCAGCTACAACTTCTGG-3′) and FPV-*VP2*-R (5′-AGGTGCGATGCATTTGACCA-3′), giving a 218 bp amplicon. Viral DNA was extracted with a commercial kit (TIANamp Virus DNA/RNA Kit, Tiangen, Beijing, China). Cycling conditions were 95 °C for 5 min; 35 cycles of 95 °C for 30 s, 56 °C for 30 s and 72 °C for 20 s; and a final extension at 72 °C for 5 min. Amplicon identity was confirmed by Sanger sequencing. For classification by DAS-ELISA, samples were scored positive when the OD450 was at least 2.1 times the mean OD450 of the negative controls on the same plate. This threshold was derived from fecal supernatants from healthy cats confirmed negative for FPV by PCR, and the number of samples in that panel together with the mean and standard deviation of their readings is given in Table S3. Agreement between the two assays is reported as the proportion of samples classified identically, that is, as concordance; diagnostic sensitivity and specificity were not determined, since that would require reference classification independent of both assays.

### 2.15. Statistical Analysis

All data are displayed as the means ± standard deviations (SDs) and were analyzed via Prism 8.1 (GraphPad, San Diego, CA, USA). One-way ANOVA tests or *t* tests were performed for statistical analysis (*p* < 0.05 was considered to indicate a significant difference).

### 2.16. Statement of Informed Consent

The owners of the animals sampled gave written consent for the samples to be used in this study.

## 3. Results

### 3.1. Virus Isolation and Identification

**Figure 1:**
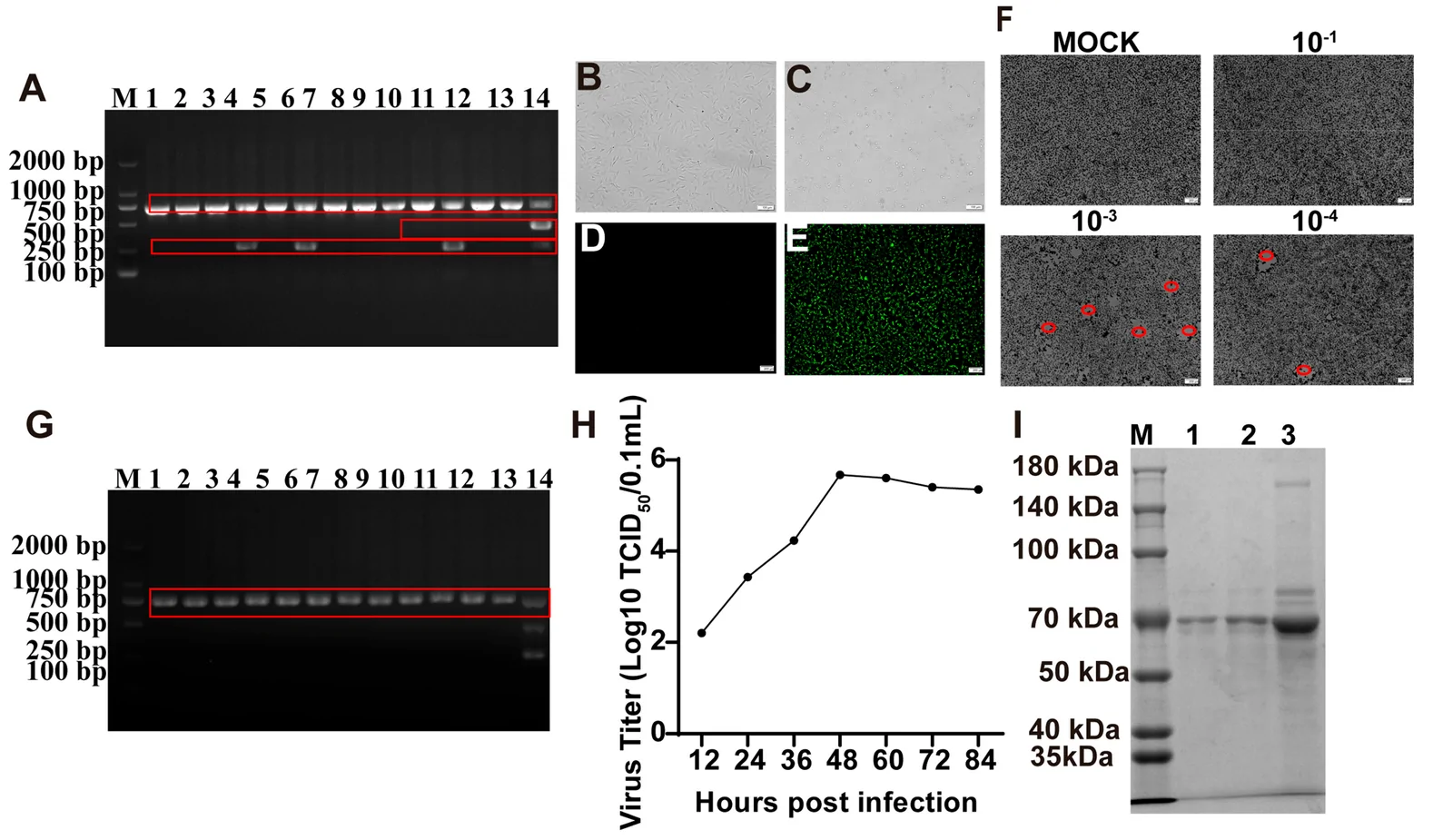
**Isolation and identification of FPV.** (**A**) Screening of clinical specimens for FCV, FPV and FHV. Lanes 1−13, clinical specimens; lane 14, positive control; M, 2000 bp DNA marker. (**B**) Uninfected CRFK cells. (**C**) CPE in CRFK cells following FPV infection. (**D**) Plaques formed by the isolate on CRFK monolayers stained with neutral red, from which single plaques were picked for three successive rounds of purification. (**E**) PCR and RT-PCR on the plaque-purified stock, showing amplification of FPV and absence of FCV; the FCV-positive clinical specimen was included as a template-positive control for the FCV assay. (**F**) Immunofluorescence of uninfected CRFK cells. (**G**) Immunofluorescence of FPV-infected CRFK cells. (**H**) One-step growth curve of FPV ZZ202501. (**I**) Verification of virus purification by Coomassie brilliant blue staining. M, 250 kDa dual-color pre-stained protein marker; lane 1, supernatant after ultracentrifugation in MEM; lane 2, fraction collected from 20% iodixanol after ultracentrifugation; lane 3, final concentrated and purified FPV suspension. Boxes indicate the target bands; circles indicate the cytopathic lesions observed in FPV-infected CRFK cells.

Clinical specimens from cats with signs consistent with feline panleukopenia were first screened for FCV, FPV and FHV. Both FCV and FPV were detected in the specimens (Figure 1A); so, any isolate obtained from them required confirmation of identity and purity. Processed specimens were inoculated onto CRFK cells, and by approximately 72 h, CPE consisting of cell shrinkage, clustering and detachment was evident (Figure 1B,C). The virus was then purified by plaque isolation: monolayers were stained with neutral red, and single plaques were picked and carried through three successive rounds of purification (Figure 1D). The purified stock was tested by PCR for FPV and by RT-PCR for FCV. FPV was amplified, whereas FCV was not, with the FCV-positive clinical specimen included in the same run as a template-positive control to confirm that the FCV assay was functional (Figure 1E); the FPV amplicon was confirmed by Sanger sequencing. Immunofluorescence of cells showing CPE gave a specific signal with the anti-FPV antibody, distributed diffusely through infected cells, while mock-infected controls showed none (Figure 1F,G). A one-step growth curve constructed from samples taken at 6, 12, 24, 36, 48 and 72 hpi showed that the titer rose from 12 hpi and plateaued by 48 hpi, reaching 10^5.67^ TCID_50_/0.1 mL (Figure 1H). SDS-PAGE of the purified preparation resolved a band at approximately 70 kDa, consistent with the predicted mass of VP2 (Figure 1I). Taken together, the PCR exclusion of FCV, the sequence-confirmed FPV amplicon and the immunofluorescence result identify the isolate as FPV, designated FPV ZZ202501.

### 3.2. Expression and Purification of the Recombinant FPV VP2 Protein

**Figure 2:**
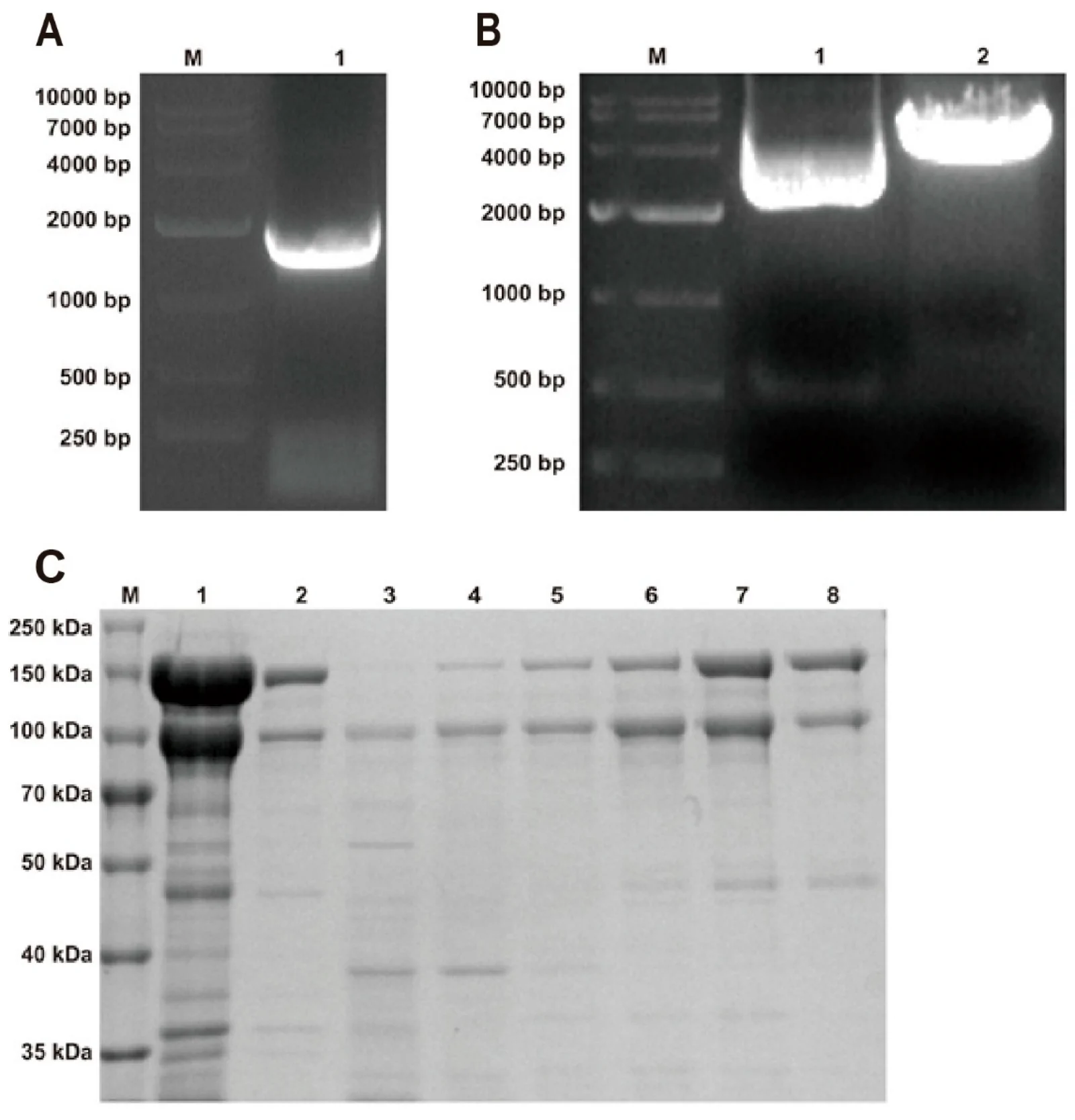
**Prokaryotic expression and purification of recombinant FPV VP2 protein.** (**A**) PCR amplification of the FPV-VP2 target gene. M, DL 10,000 DNA marker; lane 1, full-length FPV *VP2* gene (1755 bp). (**B**) Double restriction enzyme digestion of the recombinant pET-21b-NusA plasmid. M, DL 10,000 DNA marker; lane 1, intact pET-21b-NusA plasmid; lane 2, linearized plasmid fragment (5500 bp) obtained by double enzyme digestion. (**C**) Purification profile of recombinant VP2 protein via nickel affinity chromatography. M, 250 kDa dual color prestained protein marker; lane 1, flow-through fraction after supernatant-nickel binding; lane 2, residual impurities eluted with PBS washing buffer; lane 3 to lane 8, eluted fractions using gradient imidazole at concentrations of 10 mM, 25 mM, 50 mM, 100 mM, 250 mM, and 500 mM, respectively.

The FPV *VP2* gene was successfully amplified from the purified FPV genome, yielding a specific PCR product of approximately 1755 bp (Figure 2A). The VP2 fragment was successfully cloned into the pET-21b-NusA expression vector, and the recombinant plasmid pET-21b-NusA-FPV-VP2 was confirmed by sequencing (Figure 2B). Following expression in *E. coli* BL21 (DE3), the recombinant VP2 protein was successfully obtained and purified by Ni-NTA affinity chromatography. SDS-PAGE analysis revealed a distinct protein band at approximately 140 kDa, which was consistent with the expected molecular weight of the VP2-NusA fusion protein (Figure 2C). These results confirmed the successful expression and purification of recombinant FPV VP2 protein, which was subsequently used as the coating antigen for indirect ELISA-based hybridoma screening.

### 3.3. Selection and Characterization of mAbs Against Linear, Denaturation-Resistant VP2 Epitopes

**Figure 3:**
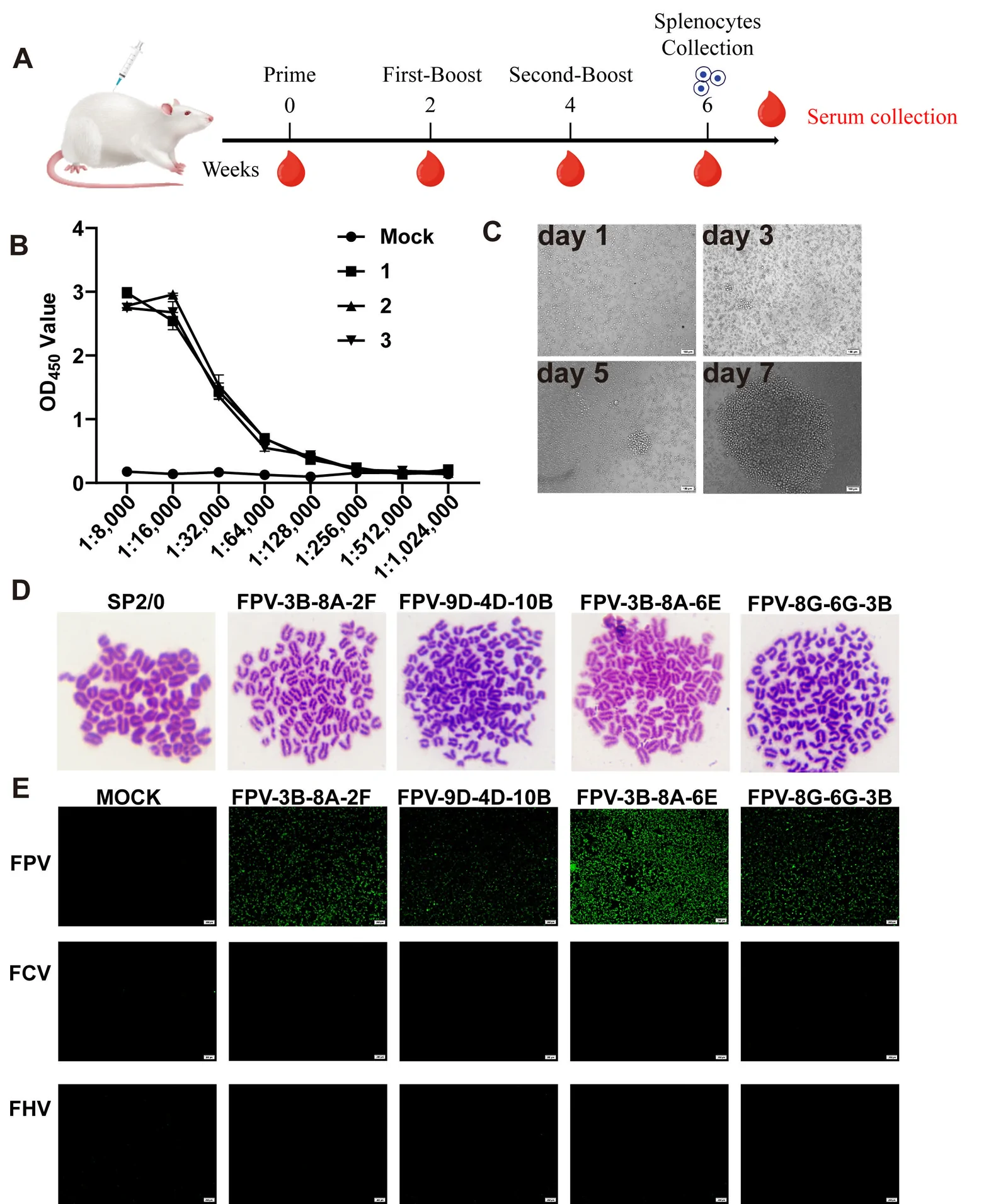
**Preparation and preliminary identification of FPV-specific monoclonal antibodies (mAbs).** (**A**) Schematic workflow of mouse immunization and hybridoma preparation. (**B**) Serum antibody titers of negative control BALB/c mice and three experimentally immunized mice determined by indirect ELISA. (**C**) Daily morphological observation and growth status of hybridoma cells after cell fusion. (**D**) Chromosome number identification of screened positive hybridoma cell lines. (**E**) Specific reactivity of prepared mAbs against FPV detected by indirect immunofluorescence assay (IFA).

BALB/c mice were immunized with concentrated, purified FPV (Figure 3A), and serum antibody responses were measured by indirect ELISA. Titers in the immunized group reached 1:128,000, well above those of the negative control group (Figure 3B), indicating a strong virus-specific response. After fusion of splenocytes with SP2/0 myeloma cells, cell clusters were visible at 3 to 4 days, and clone numbers had increased markedly by days 6 to 7 (Figure 3C). Hybridoma supernatants were screened by indirect ELISA and immunofluorescence, and four lines secreting VP2-specific antibody stably were selected and designated FPV-3B-8A-2F, FPV-9D-4D-10B (hereafter 4D10), FPV-3B-8A-6E and FPV-8G-6G-3B. Chromosome counts (Figure 3D) were 60 to 70 for SP2/0 cells and 100 to 120 for the four hybridoma lines, consistent with successful fusion. All four antibodies reacted with the prokaryotically expressed recombinant protein, and 4D10 gave a strong signal on reducing, denaturing Western blots, indicating that its epitope survives denaturation. None of the four antibodies reacted with FCV or FHV (Figure 3E).

### 3.4. Preparation and Characterization of Anti-FPV VP2 mAb Ascites

**Figure 4:**
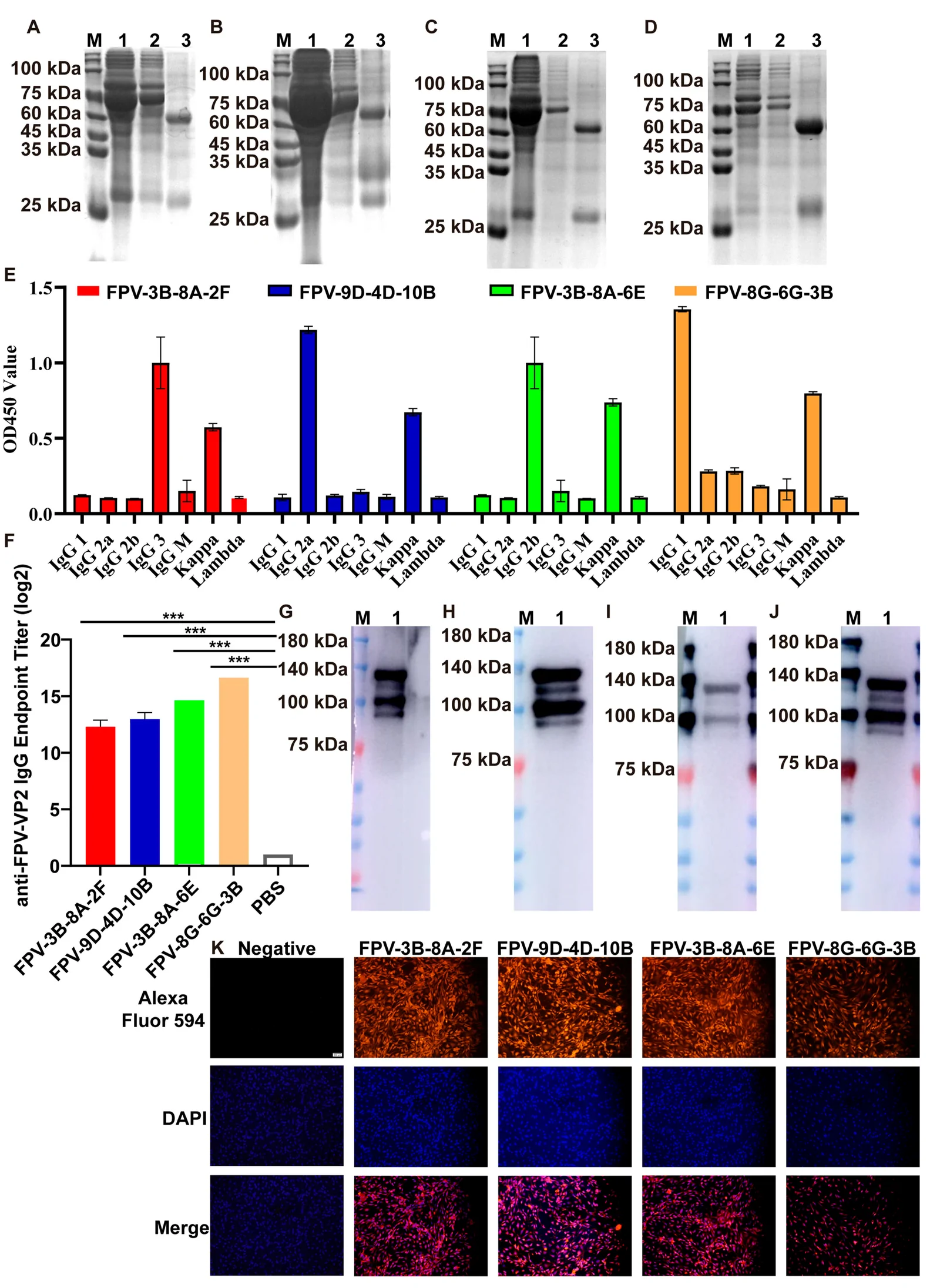
**Purification and functional validation of anti-VP2 monoclonal antibodies.** (**A**–**D**) Purity detection of monoclonal antibody purified from ascites via Coomassie brilliant blue staining. (**E**) Immunoglobulin subtype identification of screened anti-VP2 monoclonal antibodies. (**F**) Determination of effective titers of purified anti-VP2 mAbs by indirect ELISA. (**G**–**J**) Antigen-binding reactivity of different mAbs verified by Western blotting. (**K**) Specific immunoreactivity of monoclonal antibodies against native FPV verified by IFA. *** *p* < 0.0001.

SDS-PAGE of the purified antibodies resolved bands at 55 kDa and 25 kDa, corresponding to heavy and light chains, without additional bands (Figure 4A–D). Isotyping with a mouse monoclonal antibody typing kit assigned FPV-3B-8A-2F to IgG3, FPV-9D-4D-10B to IgG2a, FPV-3B-8A-6E to IgG2b and FPV-8G-6G-3B to IgG1; all four carried kappa light chains (Figure 4E). ELISA titers of the purified antibodies ranged from 1:212.64 to 1:216.64 (Figure 4F). All four reacted with FPV VP2 on Western blots (Figure 4G–J), and all gave specific signal in infected CRFK cells by immunofluorescence (Figure 4K).

### 3.5. Dynamic Expression and Subcellular Localization of the VP2 Protein

**Figure 5:**
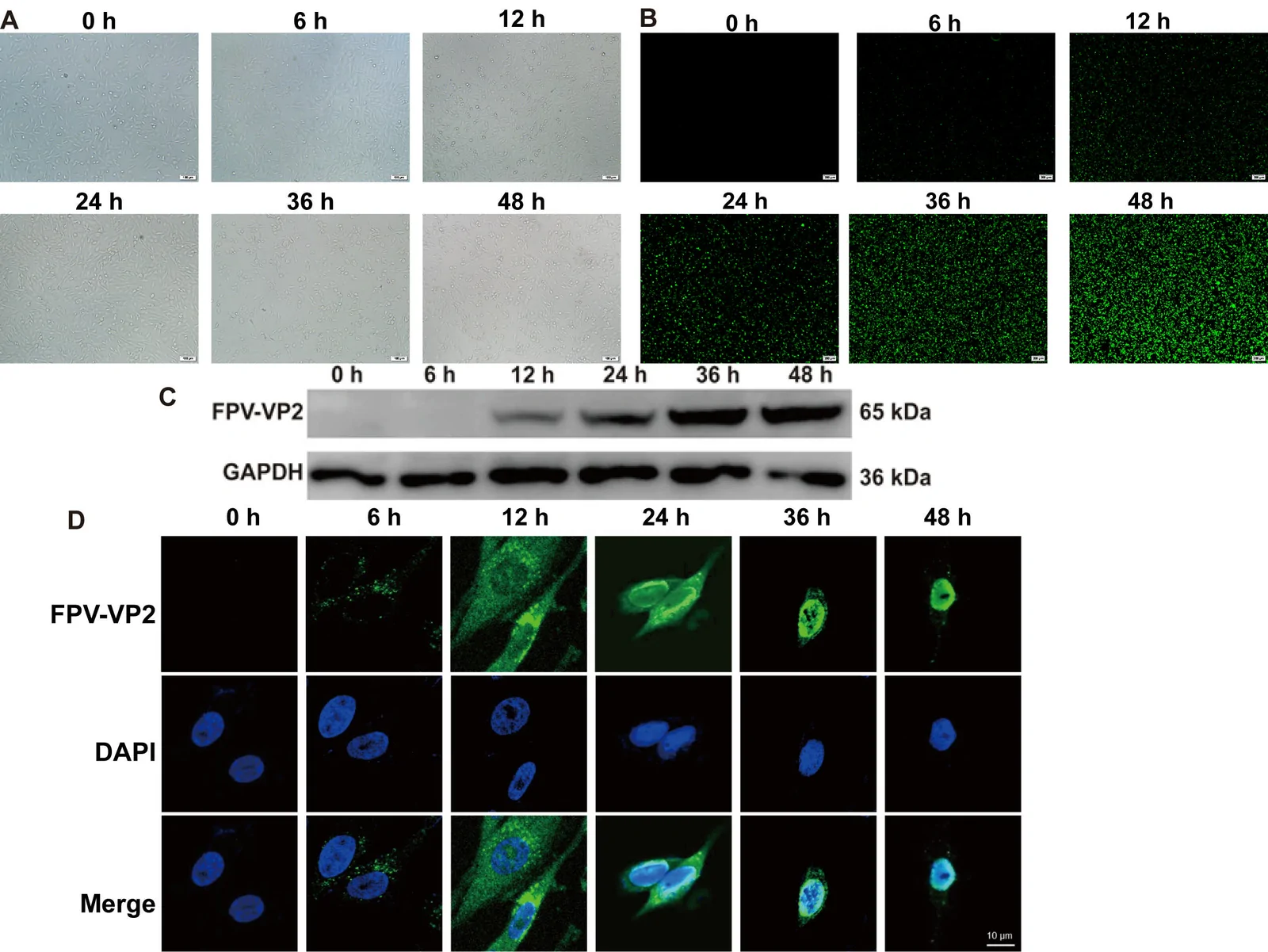
**Dynamic subcellular localization of VP2 protein in FPV-infected CRFK cells at different time points.** (**A**) Progressive CPE in CRFK cells post FPV infection. (**B**) Time-dependent expression of viral VP2 protein in CRFK cells infected with FPV strain ZZ202501 detected by IFA. (**C**) Western blot analysis of VP2 protein levels at different infection stages with GAPDH as a loading control. (**D**) Dynamic subcellular distribution characteristics of VP2 protein during FPV replication in host cells.

To follow the expression and localization of VP2 during infection, mAb 4D10 was used for both immunofluorescence and Western blotting. CPE was apparent in CRFK cells by 12 hpi, with rounding and extensive detachment by 36 to 48 hpi (Figure 5A). Specific immunofluorescence first appeared at 12 hpi, increased thereafter and was stable between 36 and 48 hpi (Figure 5B). Western blotting with GAPDH as loading control showed a time-dependent increase in VP2, with the band first detectable at 12 hpi and reaching a maximum between 36 and 48 hpi (Figure 5C). Examination of subcellular distribution at successive time points (Figure 5D) showed a small amount of VP2 in the cytoplasm as early as 6 hpi. Between 6 and 24 hpi, the cytoplasmic signal increased and accumulated in the perinuclear region, and between 24 and 48 hpi, the cytoplasmic signal declined, while nuclear signal rose and became concentrated. VP2 therefore appears first in the cytoplasm and is subsequently redistributed to the nucleus as infection proceeds.

### 3.6. Intact Microtubules Are Required for VP2 Nuclear Translocation and Efficient FPV Progeny Production

**Figure 6:**
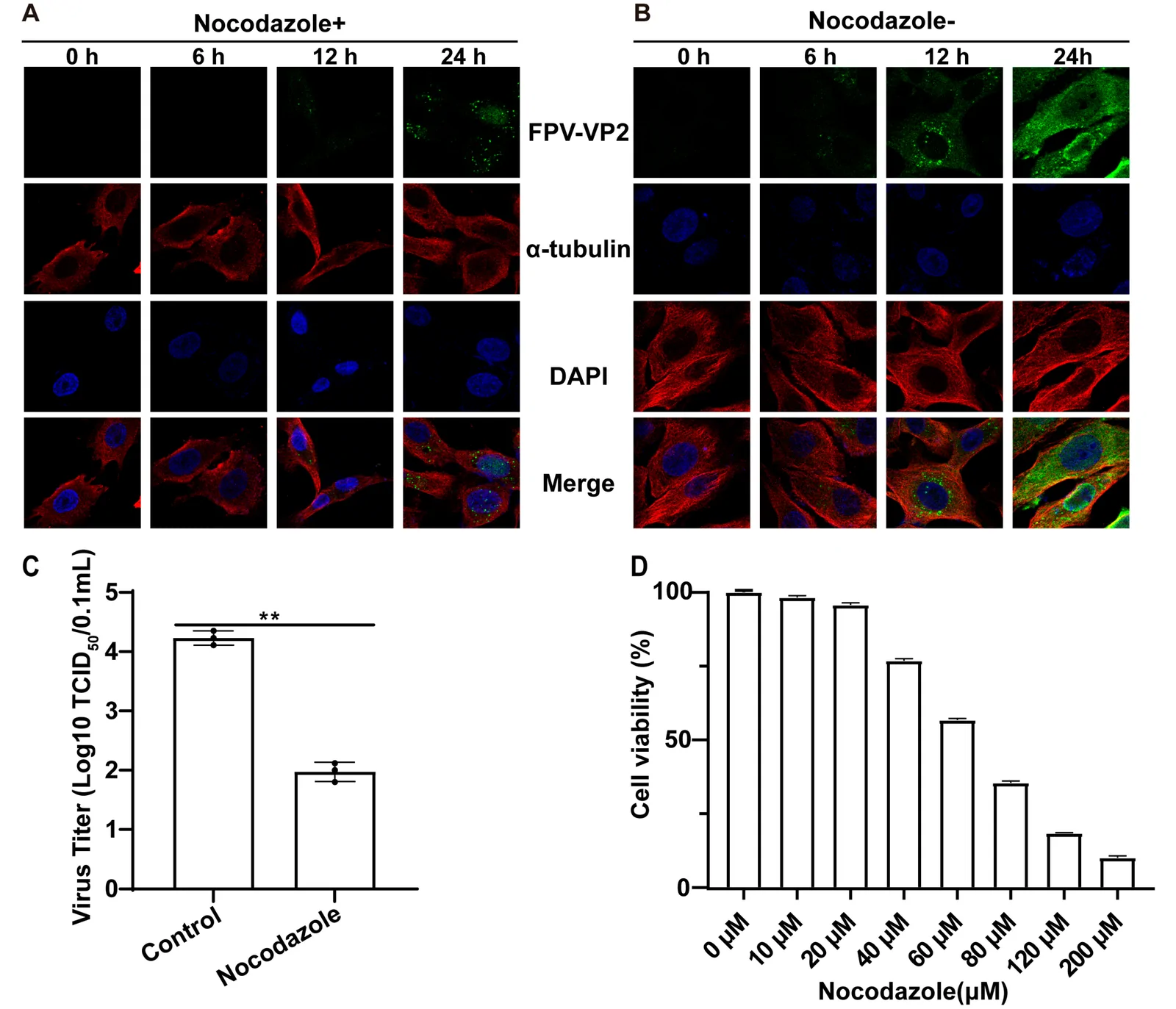
**Effects of microtubule inhibition on VP2 subcellular trafficking and FPV replication.** (**A**,**B**) CRFK cells were pretreated with 20 μM nocodazole for 4 h to block microtubule assembly, followed by infection with FPV strain ZZ202501 at an MOI of 1. The nocodazole intervention was maintained throughout the whole infection period. Cells were fixed at 0 h, 6 h, 12 h, and 24 h post-infection for immunofluorescence staining. VP2 protein was labeled with mouse anti-VP2 monoclonal antibody 4D10 (green fluorescence), the cytoskeletal microtubule structure was labeled with rabbit anti-α-tubulin antibody (red fluorescence), and cell nuclei were counterstained with DAPI (blue fluorescence). All fluorescence images were acquired using a laser confocal scanning microscope; scale bar, 10 μm. (**C**) Quantification of infectious viral titers in the supernatant of DMSO- and nocodazole-treated cells at the indicated times post-infection. (**D**) Cell viability of CRFK cells after treatment with nocodazole at the indicated concentrations for 24 h. Cell viability is expressed as a percentage of the DMSO-treated control. ** *p* < 0.01.

Microtubules support the directed transport of cargo within infected cells, and we asked whether the redistribution of VP2 depends on them. CRFK cells were treated with nocodazole to depolymerize microtubules, with DMSO as the control. Nocodazole disrupted the microtubule network (red), and VP2 (green) failed to accumulate in the perinuclear region or to enter the nucleus, remaining dispersed through the cytoplasm (Figure 6A,B). The change in distribution was accompanied by a fall in virus production: at 24 hpi, the infectious titer in the nocodazole-treated cultures was 10^2.0 ± 0.2^ TCID_50_/0.1 mL, more than 100-fold below the DMSO control at 10^4.3 ± 0.1^ TCID_50_/0.1 mL (*p* < 0.01) (Figure 6C). The viability of CRFK cells exposed to 20 μM nocodazole for 24 h did not differ significantly from DMSO-treated cells (Figure 6D); so, the reduction in titer is unlikely to be explained by the loss of viable cells. An intact microtubule network is thus required for efficient production of infectious FPV, although nocodazole also arrests cells in mitosis, and effects on the cell cycle cannot be excluded by these experiments.

### 3.7. Establishment of a Homologous Double-Antibody Sandwich ELISA

**Figure 7:**
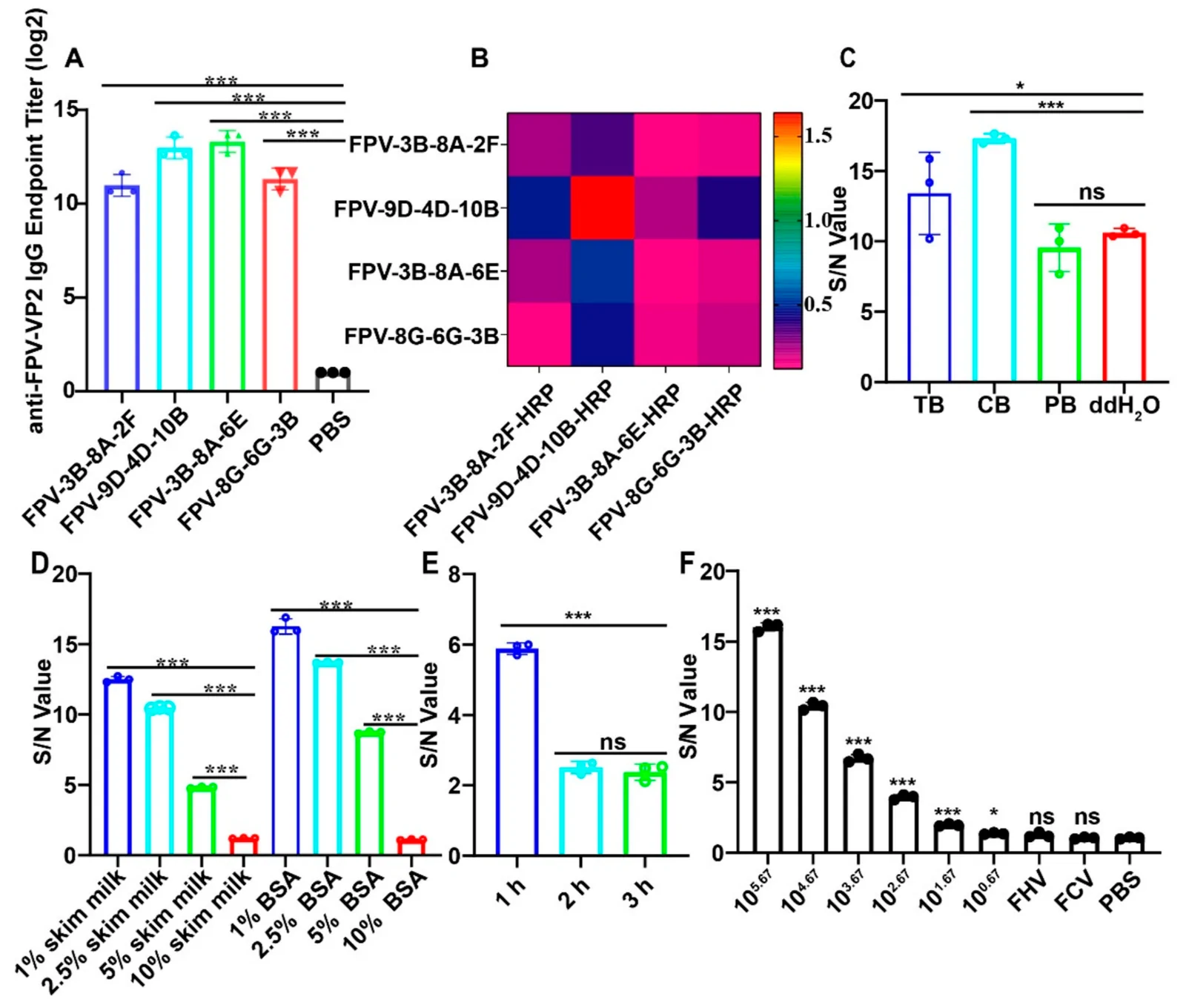
**Establishment and methodological optimization of a double-antibody sandwich ELISA for rapid FPV antigen detection.** (**A**) Working titer evaluation of monoclonal antibodies after HRP conjugation. (**B**) Screening and combination optimization of matched capture and detection antibody pairs targeting intact FPV virions. (**C**) Optimization of four commonly used coating buffer formulations for antigen immobilization. (**D**) Comparative evaluation of blocking efficiency using eight different blocking reagents: 1%, 2.5%, 5%, and 10% skim milk, as well as 1%, 2.5%, 5%, and 10% bovine serum albumin (BSA). (**E**) Optimization of incubation duration for optimal blocking performance. (**F**) Analytical specificity and detection sensitivity validation of the established FPV double-antibody sandwich ELISA method. ns *p* > 0.05, * *p* > 0.01, *** *p* < 0.0001.

After HRP conjugation, ELISA titers of the four purified mAbs ranged from 210.64 to 213.64 (Figure 7A). Checkerboard titration of all 16 capture and detection combinations showed that 4D10 used as both a capture and detection antibody gave the highest P/N ratio, 25.3 (Figure 7B). The homologous pairing therefore functions, which requires that an antigen unit bind at least two molecules of 4D10 at once; the most straightforward explanation is that the epitope is displayed repeatedly on the capsid in an orientation permitting simultaneous binding, but since the epitope was not mapped, this remains a hypothesis. Optimization identified carbonate buffer (0.1 mol/L, pH 9.6) as the coating buffer (Figure 7C), 1% BSA as the blocking agent (Figure 7D) and 1 h as the blocking time (Figure 7E). The limit of detection was 10^1.67^ TCID_50_/0.1 mL, and no signal was obtained with FHV or FCV (Figure 7F). Applied to 136 field samples, the DAS-ELISA classified every sample in the same way as the comparator PCR, giving 100% concordance (Table S3); because the comparator is an in-house assay rather than an independently validated reference, this figure describes agreement between the two assays and not the diagnostic sensitivity or specificity of the DAS-ELISA. Intra-assay and inter-assay coefficients of variation ranged from 0.8% to 6.0% (Table S2).

## 4. Discussion

Feline panleukopenia remains prevalent in China and elsewhere, and because its clinical presentation is not specific, and mixed infection with other feline pathogens is common, laboratory confirmation matters for both case management and control [26]. VP2 is an established diagnostic target: it is produced abundantly during replication, is antigenically stable and carries the dominant antigenic determinants of the virus [27,28]. This study reports two related results. An antigen-capture DAS-ELISA was built on a single monoclonal antibody directed at a denaturation-resistant epitope of VP2 and was characterized analytically, and the redistribution of VP2 to the nucleus during infection was shown to require an intact microtubule network and to be associated with efficient production of progeny virus [29].

The design rested on selecting antibodies whose epitopes survive denaturation [30]. Mice were immunized with native virus, which presents the antigen in its assembled form, but hybridomas were screened on recombinant VP2 expressed in E. coli, which lacks eukaryotic folding and post-translational modification. Screening on this antigen selects against antibodies that require native conformation and enriches for those recognizing linear denaturation-resistant determinants. The practical consequence is that the resulting assay remains reactive when capsid integrity has been lost during collection, transport and storage, conditions that fecal samples routinely encounter. This property is a plausible contributor to the complete agreement we observed between the DAS-ELISA and PCR across 136 clinical samples, although with concordant results in every sample, the data do not allow the contribution to be quantified [31,32].

Set against published serological and antigen-detection formats for FPV, the assay occupies a particular niche rather than superseding the alternatives. Most reported FPV ELISAs are indirect or dot formats that measure host anti-VP2 antibody [25,33]; these are informative about immune status but cannot establish that an animal is shedding virus. The assay described here captures viral antigen directly, with an analytical limit of detection of 10^1.67^ TCID_50_/0.1 mL. Immunochromatographic and recombinase polymerase amplification-based point-of-care tests are simpler to perform and require less equipment [34], but sensitivity at low viral load is a recognized limitation. Real-time PCR offers higher sensitivity and quantification [13], at the cost of instrumentation, reagents and trained staff that are not always available in small-animal practice [35]. A plate-based antigen-capture ELISA sits between these options: it is suited to batches of samples in a laboratory with a plate reader, and it reports antigen rather than nucleic acid or host antibody. Antigen-capture assays for canine parvovirus have been developed on the same conserved region of VP2, which is consistent with this region being a workable target across the carnivore protoparvoviruses, but also means that species-level discrimination cannot be assumed for an assay directed at it.

That mAb 4D10 works as both capture and detection antibody in a homologous sandwich is an observation; the explanation is not. The parvovirus capsid is assembled from 60 copies of the capsid protein with icosahedral symmetry; so, any surface epitope recurs many times on a single particle at separations that are large relative to the footprint of an antibody. Repeated display of this kind would account for an antigen unit binding two molecules of 4D10 at once, which is what the highest P/N ratio of the 16 combinations tested (25.3) implies. We present this as a hypothesis. Western blotting establishes only that the epitope survives reduction and denaturation; it locates no sequence and says nothing about how often the epitope occurs on an assembled capsid or how accessible it is. Overlapping peptide scanning, phage display or structural analysis would be needed to test the interpretation and to define the epitope. The VP2 coding sequence of FPV is highly conserved among reported isolates [14], which is encouraging for the prospects of broad reactivity, but conservation of the protein is not evidence that the 4D10 epitope is conserved while the epitope remains unlocated; that requires testing the antibody against antigenically distinct isolates.

The trafficking experiments bear on the cell biology of the infection. Nuclear accumulation of VP2 has been described, but whether the route to the nucleus matters for the outcome of infection was not established. Using 4D10, which detects VP2 irrespective of assembly state, we found that redistribution of the total VP2 pool to the nucleus depends on intact microtubules and that depolymerizing them reduced progeny titer by more than 100-fold, from 10^4.3^ to 10^2.0^ TCID_50_/0.1 mL, without a detectable fall in cell viability. The transport step is therefore linked to productive replication rather than incidental to it. This is consistent with reports of microtubule- and dynein-dependent movement of parvovirus capsids towards the nucleus and with evidence that the nuclear uptake of capsid protein oligomers is specified by capsid-encoded localization motifs rather than by the cytoskeleton itself [36,37]; in the autonomous parvoviruses, such motifs have been attributed principally to the unique N-terminal region of VP1 rather than to VP2. A reasonable reading of our data is that FPV uses microtubule-based transport to bring newly synthesized VP2, most probably as assembled capsids, to the perinuclear region for nuclear entry and genome packaging. The molecular link between VP2 and the microtubule track was not identified, since a depolymerizing drug cannot distinguish direct binding to tubulin from transport by a motor complex, nor can it separate the loss of transport from the mitotic arrest that nocodazole also causes.

Several limitations bear on how these results should be read. The work rests on a single isolate, ZZ202501; so, coverage of antigenically distinct FPV strains was not assessed. Analytical specificity was tested against FCV and FHV only; feline coronavirus, feline leukemia virus and other co-infecting agents were not included, and canine parvovirus was not tested; so, the assay must not be used to distinguish feline from canine parvovirus. The epitope recognized by 4D10 has not been mapped; so, the interpretation offered for the homologous pairing remains untested. The comparator for clinical testing was an in-house conventional PCR rather than a validated reference assay, and the agreement of 100% is therefore concordance between two assays and not a measure of diagnostic sensitivity or specificity; establishing those would require a larger panel of positive and negative samples characterized independently of both assays, with the panel defined in advance. The cut-off was derived from a small panel of confirmed negative fecal supernatants and should be re-derived by receiver operating characteristic analysis on a larger panel before clinical use. Because the comparator was read on agarose gels, viral loads in the field samples were not quantified; so, performance near the limit of detection has not been established. Finally, the trafficking work used one pharmacological inhibitor and did not identify the motor or adaptor involved. Mapping the 4D10 epitope, testing the assay against a wider panel of feline viruses and FPV isolates, and identifying the transport machinery are the next steps.

## Data Availability

The original contributions presented in this study are included in the article/Supplementary Material. Further inquiries can be directed to the corresponding authors.

## Supplementary Materials

The following supporting information can be downloaded at https://www.mdpi.com/article/10.3390/microorganisms14102412/s1, Table S1: Primer Sequen. Table S2: Repetitive test result. Table S3: Comparison between double-antibody sandwich ELISA and conventional PCR detection methods. Table S4: The optimal coating concentration of FPV-VP2 protein.
