# Supplementary material for: Homologous Double-Antibody Sandwich ELISA Targeting a Linear Epitope of FPV VP2 and Demonstration of Microtubule-Dependent Intracellular Trafficking
Source: Microorganisms. 2026 Oct 21;14(10):2412. doi: 10.3390/microorganisms14102412 (PMC13707527; doi:10.3390/microorganisms14102412)
Supplement: Supplementary file 1 [file microorganisms-14-02412-s001.zip › Supplementary Table S1.pdf]

Supplementary Table S1 Primer Sequen

Primer name	Primer sequence(5´-3´)	Length(bp)	temperature(℃)
FPV-*VP2*-JD-F	ACGGGTCTGGAGGCGGGGGTGGTG	713	56
FPV-*VP2*-JD-R	AGCAAATTCATCACCTGTTCTTA		
FHV-*gD*-F	TGGTGTCGAAATGTTAAAACTAT	441	56
FHV-*gD*-R	TGCAGTTGATCGAGACCTCTTTA		
FCV-*VP1*-F	TGCCGTTGGTGGTGTGATTGCT	258	56
FCV-*VP1*-R	CCCGAGATAGAAAAGCGAACAT		
FPV-VP2-F	GGAACTAGTGGCACACCAACA	1755	56
FPV-VP2-R	GGCCCTTGTGTAGACGCTT		
