# Supplementary material for: Homologous Double-Antibody Sandwich ELISA Targeting a Linear Epitope of FPV VP2 and Demonstration of Microtubule-Dependent Intracellular Trafficking
Source: Microorganisms. 2026 Oct 21;14(10):2412. doi: 10.3390/microorganisms14102412 (PMC13707527; doi:10.3390/microorganisms14102412)
Supplement: Supplementary file 1 [file microorganisms-14-02412-s001.zip › Supplementary Table S2.pdf]

Supplementary Table S2 Repetitive test result

	Intra-assay CV		Inter-assay CV	
	X±SD	CV(%)	X±SD	CV(%)
Positive samples	1.429± 0.036	2.73%	1.37± 0.035	3.06%
	1.401± 0.016	1.27%	1.36± 0.097	7.77%
	1.472± 0.076	5.98%	1.32± 0.059	4.82%
Negative samples	0.106± 0.004	4%	0.137± 0.006	4.8%
	0.105± 0.008	0.8%	0.134± 0.004	3.36%
	0.122± 0.002	2.5%	0.148± 0.001	1.34%
