# Supplementary material for: Homologous Double-Antibody Sandwich ELISA Targeting a Linear Epitope of FPV VP2 and Demonstration of Microtubule-Dependent Intracellular Trafficking
Source: Microorganisms. 2026 Oct 21;14(10):2412. doi: 10.3390/microorganisms14102412 (PMC13707527; doi:10.3390/microorganisms14102412)
Supplement: Supplementary file 1 [file microorganisms-14-02412-s001.zip › Supplementary Table S3.pdf]

Supplementary Table S3 Comparison between double-antibody sandwich ELISA and conventional PCR detection methods

Sample type	Comparative analysis	
	Conventional PCR	DAS-ELISA
Positive sample	47	47
Negative sample	89	89
Positive rate	34.56%	34.56%
