# Supplementary material for: Homologous Double-Antibody Sandwich ELISA Targeting a Linear Epitope of FPV VP2 and Demonstration of Microtubule-Dependent Intracellular Trafficking
Source: Microorganisms. 2026 Oct 21;14(10):2412. doi: 10.3390/microorganisms14102412 (PMC13707527; doi:10.3390/microorganisms14102412)
Supplement: Supplementary file 1 [file microorganisms-14-02412-s001.zip › Supplementary Table S4.pdf]

Supplementary Table S4. The optimal coating concentration of FPV-VP2 protein

Serum dilution	antigen concentration (ng/mL)					
		800	400	200	100	50
1:400	Positive serum OD_{450}	1.65	1.46	1.63	1.58	1.67
	Negative serum OD_{450}	0.09	0.06	0.06	0.06	0.09
	P/N	18.33	24.33	27.17	26.33	18.56
1:800	Positive serum OD_{450}	2.06	2.07	2.51	2.04	2.73
	Negative serum OD_{450}	0.07	0.07	0.08	0.31	0.05
	P/N	29.43	29.57	31.38	6.58	**54.6**
1:1 600	Positive serum OD_{450}	1.99	2.37	2.85	2.13	2.74
	Negative serum OD_{450}	0.08	0.06	0.06	0.05	0.57
	P/N	24.88	39.5	47.5	42.6	4.81
1:3 200	Positive serum OD_{450}	2.28	2.36	2.30	2.53	2.46
	Negative serum OD_{450}	0.08	0.06	0.06	0.05	0.09
	P/N	28.5	39.33	38.33	50.60	27.33
1:6 400	Positive serum OD_{450}	1.75	1.28	1.10	1.32	1.15
	Negative serum OD_{450}	0.06	0.05	0.05	0.12	0.05
	P/N	29.17	25.60	22.00	11.00	23.00
1:12 800	Positive serum OD_{450}	1.23	1.28	1.10	1.32	1.15
	Negative serum OD_{450}	0.07	0.07	0.06	0.05	0.06
	P/N	17.57	18.29	18.33	26.40	19.17
1:25 600	Positive serum OD_{450}	1.15	0.68	0.65	1.17	0.64
	Negative serum OD_{450}	0.07	0.08	0.06	0.07	0.06
	P/N	16.43	8.50	10.83	16.71	10.67
1:51 200	Positive serum OD_{450}	0.48	0.28	0.41	0.64	0.40
	Negative serum OD_{450}	0.11	0.12	0.09	0.09	0.12
	P/N	4.36	2.33	4.56	7.11	3.33
